# GEMDiff: a diffusion workflow bridges between normal and tumor gene expression states: a breast cancer case study

**DOI:** 10.1093/bib/bbaf093

**Published:** 2025-03-11

**Authors:** Xusheng Ai, Melissa C Smith, F Alex Feltus

**Affiliations:** Department of Electrical and Computer Engineering, Clemson University, Clemson, SC 29634, United States; Department of Electrical and Computer Engineering, Clemson University, Clemson, SC 29634, United States; Department of Genetics and Biochemistry, Clemson University, Clemson, SC 29634, United States; Biomedical Data Science and Informatics Program, Clemson University, Clemson, SC 29634, United States; Center for Human Genetics, Clemson University, Clemson, SC 29634, United States

**Keywords:** generative model, diffusion model, gene state transition, genotype–phenotype interaction, genetic subsystem discovery

## Abstract

Breast cancer remains a significant global health challenge due to its complexity, which arises from multiple genetic and epigenetic mutations that originate in normal breast tissue. Traditional machine learning models often fall short in addressing the intricate gene interactions that complicate drug design and treatment strategies. In contrast, our study introduces GEMDiff, a novel computational workflow leveraging a diffusion model to bridge the gene expression states between normal and tumor conditions. GEMDiff augments RNAseq data and simulates perturbation transformations between normal and tumor gene states, enhancing biomarker identification. GEMDiff can handle large-scale gene expression data without succumbing to the scalability and stability issues that plague other generative models. By avoiding the need for task-specific hyper-parameter tuning and specific loss functions, GEMDiff can be generalized across various tasks, making it a robust tool for gene expression analysis. The model’s ability to augment RNA-seq data and simulate gene perturbations provides a valuable tool for researchers. This capability can be used to generate synthetic data for training other machine learning models, thereby addressing the issue of limited biological data and enhancing the performance of predictive models. The effectiveness of GEMDiff is demonstrated through a case study using breast mRNA gene expression data, identifying 307 core genes involved in the transition from a breast tumor to a normal gene expression state. GEMDiff is open source and available at https://github.com/xai990/GEMDiff.git under the MIT license.

## Introduction

Breast cancer persistently poses a global public health dilemma with an estimated $2.3$ million new cases and more than 685 000 deaths reported in 2020 [1]. Although survival rates have markedly improved over the past two decades, the incidence of this disease continues to rise worldwide [2]. Breast cancer results from genetic and epigenetic mutations in normal breast tissue. Tumor aggressiveness is heavily determined by intrinsic complex gene expression patterns in tumorigenic tissue, often with unique clonal complex patterns within a heterogeneous tumor mass [3]. Given this complexity, there is an extreme need for stronger algorithms to identify the genetic underpinnings (i.e. core gene sets) driving the transition from normal breast tissue into a tumor state, which can help with cancer diagnostics, prognostics, and treatment solutions. Fortunately, deep learning models transform complex perturbational phenomena into algorithmically tractable tasks, formulating predictions based on various types of datasets [4].

All complex traits are under the control of multiple discrete genes combining in concert over space and time to express a phenotype ranging from a cellular (e.g. differentiated epithelial cell) to organismal (e.g. opposable thumb). Given the current estimate of 78 724 known genes in humans (www.gencodegenes.org/human/stats_47), it is clear that endless blends of genes could converge to drive a functional gene expression system. Our goal is to identify better approaches to detect the causal genetic subsystem underlying a specific trait. To achieve this goal, our approach is to observe and learn the gene expression pattern shifts, typically at the RNA level, between relevant states including normal to tumor transitions [5–7].

Thanks to an untold number of scientific experimentations, gene-phenotype associations have been mapped for decades using genetic and other techniques. Using genetic approaches, for example, there are now 3451 genes associated with a single phenotype and 7546 phenotypes with evidence for a specific molecular basis in the Online Mendelian Inheritance in Man (OMIM) database. Another example of gene-phenotype association prior knowledge is the human Molecular Signatures Database (MSigDB) dataset. Typically using manual curation of genomic data, MSigDB consists of 34 836 gene sets associated with thousands of cellular and organism level phenotypes. Even with all these prior clues, the relevant gene expression blends that drive complex traits are elusive.

Generative artificial intelligence has shown significant potential in assisting the cancer diagnosis domain [8]. Previously, generative adversarial networks (GANs) have been utilized to solve data augmentation problems [9] and to identify biomarkers [10–12]. Despite their success, GAN models often require a target-specific loss function to maintain training stability, which limits their generalizability across different genetic tasks [11, 13].

Diffusion models (DMs) are powerful tools in the computer vision area [14–16], exhibiting stability and high sample quality. DMs have garnered increasing attention in bioinformatics research [17–19]. As a step-by-step generative model, DMs facilitate the easy retrieval of intermediate data, providing unique insights into the transformation processes between data distributions. These models have proven useful in data augmentation and even in the imputation of missing RNA data [20–22] but preserved the U-net model, initially designed for image processing, to learn the reverse diffusion process. The applications of DMs in transforming gene states remain relatively unexplored.

**Figure 1:**
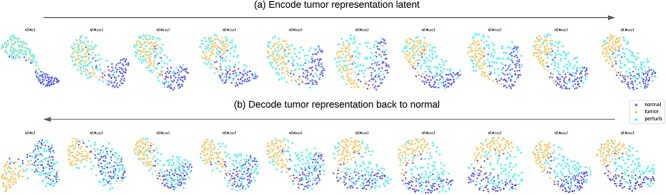
Diffusion gene perturbation with mitotic spindle gene subset: (a) given a tumor gene expression matrix, the diffusion model ran a forward ODE process to convert it to the tumor latent gene expression, while then (b) the diffusion model ran a reversed ODE process to recover the normal gene expression from the tumor latent gene expression. The number of tumor samples was greater than normal samples. This figure only shows the balanced number of tumor-normal samples to better visualize the perturbation process. Supplemental Fig. S1 shows the unbalanced plot.

Gene mutations that increase the fitness of cancer cells raise the odds of developing breast cancer [23], but not all mutations occur simultaneously. Inspired by DMs, which are designed to learn how to transfer between two data distributions, we propose GEMDiff—a diffusion workflow that bridges the normal and tumor transcriptome states measured in an RNA gene expression matrix (GEM). GEMDiff gradually perturbs tumor gene expression back to normal in a reverse mutation process that, if mimicked in a patient, could return tumor cells to a normal state. Figure 1 shows the diffusion breast gene perturbation, gradually transforming tumor gene expression state back to normal gene expression state.

GEMDiff produces perturbed data that match the distribution of the target state more accurately than our previous work—perturbed data from GANs tend to cluster centrally rather than reflect the true distribution of new states [11]. Thus, GEMDiff enhances the potential for new biomarker identification and addresses the scalability and stability issues inherent in GAN models. Furthermore, we have replaced the U-net model [24] with a transformer-based model [25]. This adaptation shifts the focus from neighborhood dependencies, which are typical in convolutional networks, to broader gene feature relationships. The transformer-based model is able to consider global relationships, particularly to immediate neighbors, making it a more effective tool for revealing intricate gene interrelationships than traditional convolutional networks. This improves our understanding of the genetic mechanisms that underlie cancer.

Herein we used breast cancer bulk RNAseq data as a case study. We visualize the results with UMAP [26] and validated results in a biological way—Gene Function enrichment analysis to examine the biology of the core genes. GEMDiff is open source https://github.com/xai990/GEMDiff.git and generalized to any GEM measured for two labeled phenotypes.

## Methods

### Datasets and pre-processing

**Figure 2:**
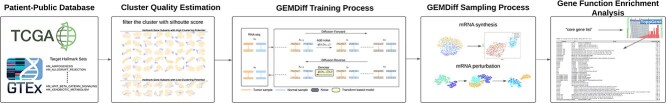
Model pipeline: (i) data processing: includes replacing the NA values, applying $log_{2}(n+1)$ transformation, and normalization; (ii) cluster quality assessment: examined by silhouette scores to selected well-clustered gene sets; (iii) model training: utilizes selected gene sets for training the diffusion model; (iv) gene augmentation/gene perturbation: implemented data augmentation or gene perturbation according to the task types and employed UMAP plots for outcome display; (v) evaluation: validate the core genes with gene function enrichment analysis.

Figure 2 shows an overview of the model workflow. First, we obtained a unified bulk RNAseq GEM derived from normal breast (brcarsemfpkmtcga.txt.gz, breastrsemfpkmgtex.txt.gz), breast tumor (brcarsemfpkmtcgat.txt.gz), norrmal thyoid (thyroidrsemfpkmgtex.txt.gz), and thyroid tumors (thcarsemfpkmtcgat.txt.gz) RNAseq samples from [27]. RNA expression was quantified in FPKM units. All samples had been quantile normalized and corrected for batch effects from the Cancer Genome Atlas (TCGA) [28] and the Genotype-Tissue Expression (GTEx) databases [29]. The breast GEM comprised $1181$ samples each with $19\ 738$ gene features. We separated the data set with a training dataset $67\%$ (788 samples) and a testing data $33\%$ (393 samples). Initially, the dataset underwent a $\log _{2}n$ transformation.

To fit the data for GEMDiff, we modified the transformation from $\log _{2}n$ to $\log _{2}(n + 1)$ to keep track of the unexpressed genes. This adjustment ensured that unexpressed genes, originally represented by negative infinity, were instead set to zero. To further refine the data, we normalized the gene set using a min-max scaler and adjusted the range to (-1, 1), thus optimizing the GEM for subsequent analysis.

### Cluster quality

We quantified the cluster quality with the silhouette score [30], which was calculated for each gene set representation. Directly applying the silhouette score to the original high-dimensional gene expression data would be computationally intensive and resource-demanding. We sidestep this issue by implementing the silhouette score as 2D representations obtained from a uniform manifold approximation and projection for dimension reduction (UMAP) that reduced the data dimensionality while preserving its global and local structure [26].


(1) \begin{align*}& s(i) = \frac{b(i)- a(i)}{max(a(i),b(i))}\end{align*}

where $b(i)$ is the distance between $i$ and its nearest cluster centroid and $a(i)$ is the average distance between $i$ and all of the others in its own cluster. The silhouette score ranges from -1 to 1, with higher values indicating better cluster separation.

### Diffusion model

Then, we design the diffusion process following the denoising diffusion probabilistic model (DDPM) [14]—note that we intentionally kept the original parameter notation in these equations to allow easily cross-reference and dive deeper into the original sources if needed. During the forward process $q(x_{1:T}| x_{0})$, noise is gradually added to the input gene expression data $x_{0}$ at each time step $t$ (Equation 2), eventually a Gaussian noise.


(2) \begin{align*}& \begin{split} q(x_{1:T}| x_{0}): = \prod_{t = 1}^{T}q(x_{t}| x_{t- 1}),\\ q(x_{t}| x_{t- 1}): = {\mathcal{N}}\left(\sqrt{\frac{\alpha_{t}}{\alpha_{t - 1}}}x_{t- 1},\left(1- \frac{\alpha_{t}}{\alpha_{t - 1}}\right)I\right) \end{split}\end{align*}

where $\alpha _{1},..., \alpha _{T}$ are scheduled decreasing weights, $x_{1},...,x_{t}$ are the noisy gene expression data. We express $x_{t}$ as a linear combination of $x_{0}$ and a noise variable $\epsilon $ due to the property of the Markov chain:


(3) \begin{align*}& x_{t} = \sqrt{\alpha_{t}}x_{0} + \sqrt{1 - \alpha_{t}}\epsilon,\,where\,\epsilon \sim{\mathcal{N}}(0,I)\end{align*}

The reverse process learns to generate target gene expression data by progressively denoising a Gaussian noise.


(4) \begin{align*}& p_{\theta}(x_{0:T}): = p_{\theta}(x_{T})\prod_{t = 1}^{T}p_{\theta}^{(t)}(x_{t - 1}| x_{t})\end{align*}

where $\theta $ is learnable model parameters to fit the gene expression data distribution $q(x_{0})$ by minimizing the objective function:


(5) \begin{align*}& L_{{\mathrm{simple}}}(\theta ): = {\mathbb{E}}_{t,x_{0},\epsilon}[ \parallel \epsilon - \epsilon_{\theta}(\sqrt{\bar{\alpha}_{t}}x_{0} + \sqrt{1 - \bar{\alpha}_{t}}\epsilon,t)\parallel^{2}]\end{align*}

Song et al. [15] generalize diffusion processes to non-Markovian that has the same marginal densities as the Markov chain diffusion such that consider both $x_{t}$ and $x_{0}$ to obtain the reverse sample $x_{t - 1}$. The model $f_{\theta }^{(t)}$ predicts the $x_{0}$ from $x_{t}$


(6) \begin{align*}& f_{\theta}^{(t)}(x_{t}): = (x_{t} - \sqrt{1 - \alpha_{t}} \cdot \epsilon_{\theta}^{(t)}(x_{t}))/ \sqrt{\alpha_{t}}\end{align*}

where combining Equations 3 and 6 resulting in the implicit form where $x_{t-1}$ is conditional on both $x_{t}$ and predicted $x_{0}$.


(7) \begin{align*}& \begin{split} x_{t - 1} = \sqrt{\alpha_{t - 1}}(\frac{x_{t} - \sqrt{1 - \alpha_{t}}\epsilon_{\theta}^{(t)}(x_{t})}{\sqrt{\alpha_{t}}}) \\ + \sqrt{1 - \alpha_{t - 1} - \sigma_{t}^{2}} \cdot \epsilon_{\theta}^{(t)}(x_{t}) + \sigma_{t}\epsilon_{t} \end{split}\end{align*}

In a special case when $\sigma _{t} = 0$ for all $t$, the diffusion process is represented by a deterministic ordinary differential equation (ODE), which enables uniquely identifiable encoding [16]. Su et al. [31] implemented ODESolve during the forward and reverse process to obtain the conversion between data and their latents (Equation 8).


(8) \begin{align*}& \begin{split} x^{(l)} = ODESolve(x^{(s)};model_{\theta}^{(s)},0,T) \\ x^{(t)} = ODESolve(x^{(l)};model_{\theta}^{(t)},T,0) \end{split}\end{align*}

where $x^{(s)}$ represents the data from source domain, $x^{(l)}$ is the encoding latent, generating by source $model_{\theta }^{(s)}$, $x^{(t)}$ is the results in the target domain, generating by target $model_{\theta }^{(t)}$ from $x^{(l)}$.

### GEMDiff model: training and sampling

**Figure 3:**
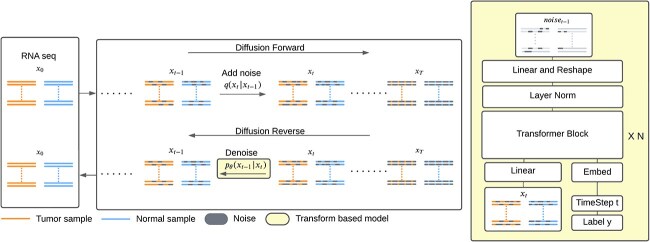
Diffusion process with RNAseq data synthesis: the colors are used to represent the feature representation: orange stands for tumor state, and blue stands for the normal state. During the diffusion forward process, the RNA seq gradually loses the tumor feature representation as the noise is added, shown in grey. By timestep T, both tumor and normal state sequences converge to a standard Gaussian distribution. In the reverse process, the model denoises the data from this Gaussian state and restores the state-specific feature representations.

Figure 3 shows the forward and reverse process for training. During the forward process, random noise was added to the RNAseq data, converging to a standard Gaussian distribution at time step $T$. To learn the reversed diffusion process (Equation 5), we utilized a transformer-based model as the main architecture [25], shown in the right side of Fig. 3. The model begins with a linear projection layer that maps genes to a higher-dimensional embedding. Then, the time step and position embedding are added, and x$N$ transformer blocks within the transformer encoder are added. A final linear projection layer reverts the embedding to the original data shape. The transfer-based model is designed to learn the noise added to the data at each diffusion step and to predict $x_{t-1}$ based on $x_{t}$.

For the gene expression augmentation task, GEMDiff generates diverse synthesized data from normal Gaussian noise data. The sampling process remains the same as that of the DDPM [14]. Instead, we focus on the quality of the synthesis among a different range of gene expression features. The feature sizes of gene expression vary according to the biology tasks required.

For the gene perturbation task, we applied the ODE sampling strategy to first obtain the latent representation of tumor gene expression $Z_{T} \in{\mathbb{R}}^{n \times f}$ from $X_{tumor} \in{\mathbb{R}}^{n \times f}$, where $n$ represents the sample number and $f$ represents the gene expression feature dimension. Then, GEMDiff sampled normal gene expression $X_{normal} \in{\mathbb{R}}^{n \times f}$ based on $Z_{T}$ (Algorithm 1). This sampling strategy ensures the maintenance of entropy-regularized optimal transport [31].




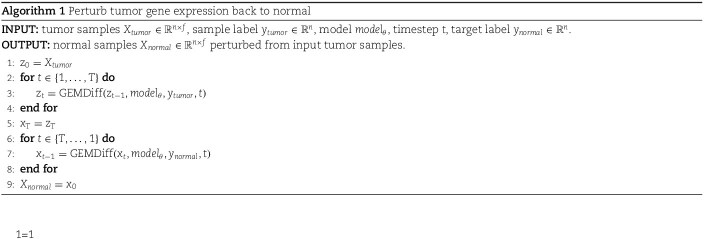



### UMAP visualizations

In our UMAP plots visualization, two key parameters are adjusted: $n\_neighbors$ and $min\_dist$. $n\_neighbors$ balances the representation of local and global structure within the data. Lower values prompt UMAP to prioritize local at the expense of global detail, while higher values emphasize global patterns over local structure. The $min\_dist$ parameter dictates the minimum separation between points in the low-dimensional space, with smaller values resulting in tighter clusters. Given UMAP’s sensitivity to global data structures, if normal and tumor sample numbers are unbalanced, the UMAP plots lose detailed information about the data with fewer samples. We set a control experiment to ensure an equal number of normal and tumor samples to better preserve the distinct local structures within each class during the perturbation process.

### M‌MD distance

Maximum mean discrepancy (MMD) is a statistical measure for comparing two distributions based on samples drawn from each distribution [32]. MMD is particularly effective in scenarios where the goal is to assess the divergence between datasets. MMD measures the distance between the mean embedding of the samples from each distribution in a reproducing kernel Hilbert space leveraging the properties of kernel functions to map data into a higher-dimensional space where the differences between distributions can be more easily distinguished. We used a Gaussian kernel:


(9) \begin{align*}& k(x,y) = exp\left( - \frac{ \parallel x - y\parallel^{2}}{2\sigma^{2}}\right)\end{align*}

where $\sigma $ determines the scale of exponential decay based on the Euclidean distance between $x$ and $y$.

### SVM classifier

Support vector machine (SVM) is a supervised learning algorithm used for classification and regression tasks [33]. SVM aims to find the optimal hyperplane that maximally separates different classes in a high-dimensional space by transforming the input space using kernel functions. We utilized SVM to classify tumor and normal samples based on the input RNA-seq gene expression data.

### Gene function enrichment analysis

Genes were input into the ToppFunn function enrichment server using their API (https://toppgene.cchmc.org/API; [34]). First official gene symbols (e.g. BRCA1) were converted NCBI gene identifiers (e.g. 672) (https://toppgene.cchmc.org/API/lookup) and then a gene set was tested for annotation term enrichment using a default gene background (https://toppgene.cchmc.org/API/enrich). The significance threshold was set at Benjamini–Yekutieli false discovery rate (BY FDR) q-value less than 1e-10.

## Results

We present three experiments to demonstrate the effectiveness of our workflow in detecting gene features involved in normal and tumorigenic human breast tissue. First, we determined appropriate UMAP visualization parameters and a cluster scoring system for data dimensionality reduction. Next, we tested our model for data augmentation and optimal gene feature size determination. Finally, we applied our model to identify genes involved in normal breast and tumor gene expression state transitions.

### UMAP plots and cluster scoring system for data dimensionality reduction

Our input GEM was labeled with normal and tumor samples from the GTEx and TCGA normal bulk RNAseq datasets. Given the high dimensionality of the dataset ($n=19\ 738$ genes), we implemented UMAP visualization to reduce high-dimensional gene expression data to 2D representations. To cluster normal and tumor gene expression, we experimented with a range of $n\_neighbors$ ($15-180$) and $min\_dist$ ($0.1-0.9$) combinations (shown in Supplemental Fig. S2). We selected $n\_neighbors = 90$ and $min\_dist = 0.3$ to represent generalized local and global structures for RNA data. Cluster quality was quantified using the silhouette score of the UMAP 2D plot with these parameters.

### GEMDiff for data augmentation

Evaluating the quality of generative models is a challenging task [35]. We employed three evaluation criteria commonly used in RNAseq data analysis: UMAP, MMD, and classification performance (SVM classifier). These three metrics serve as both quantitative and qualitative measures to comprehensively evaluate the quality of the RNAseq synthesized by our GEMDiff model.

**Figure 4:**
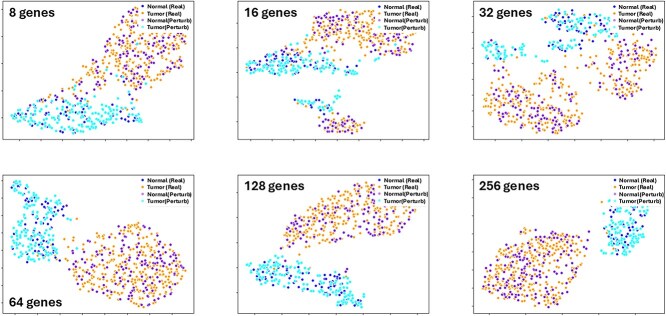
Sample clustering with gene augmentation: GEMDiff perturbation results varying ranges of gene features from 8 to 256 visualized by UMAP plots.

We evaluated the synthesis capabilities of our model across a diverse range of gene feature sizes from 8 to 256 genes. Gene sets were randomly selected from a total of 19 738 genes from the input GEM. Figure 4 shows the effect of random feature length on normal and tumor clustering. Notably, synthesis data not only aligned well with the real data but also preserved the local structure.

Table 1 shows the evaluation metrics for data augmentation using the GEMDiff model, comparing its performance with a baseline. We set the baseline by calculating classification accuracy on the test RNAseq data and computing the MMD score between the test RNAseq data and the train RNAseq data. Across all gene feature range (8, 16, 32, 64, 128, and 256), synthetic data generated by the GEMDiff consistently demonstrated performance comparable to the baseline in terms of both the MMD score (comparing synthetic data model with test data) and classification accuracy. There were exceptions when the gene features are 8 and 16. While the MMD score remains at a small value, the accuracy drops significantly compared to the baseline. This is because we trained an SVM as the classifier when the gene feature is 8 or 16. Randomly selected genes might not have a strong correlation with breast cancer, which could make it difficult for the classifier to identify the correct label for the synthesized data. However, the UMAP plots and MMD scores provide strong evidence that the synthetic data generated by the GEMDiff model follow a distribution similar to that of the real data. The low MMD scores indicate a high degree of similarity between the synthetic and real data distributions, while the UMAP plots visually demonstrate the overlap between the two datasets in the reduced-dimensional space. These results validate the effectiveness of the GEMDiff model in capturing the underlying structure and patterns present in the real data.

**Table 1:** Evaluation metrics for data augmentation with GEMDiff model

**Gene feature**	**Baseline**		**GEMDiff (100%)**		**GEMDiff (50%)**		**GEMDiff (30%)**	
	MMD score	Accuracy	MMD score	Accuracy	MMD score	Accuracy	MMD score	Accuracy
8	5.29E-5	81.17%	4.22E-3	47.00%	4.20E-3	47.67%	3.71E-3	50.00%
16	2.74E-4	86.51%	4.85E-3	80.00%	6.08E-3	52.00%	5.99E-3	69.67%
32	2.79E-4	93.13%	6.17E-3	94.00%	8.52E-3	89.67%	9.60E-3	86.33%
64	2.79E-4	93.64%	5.19E-3	97.67%	4.17E-3	91.00%	6.01E-3	86.00%
128	2.87E-4	97.46%	4.41E-3	98.67%	6.09E-3	98.67%	8.25E-3	99.00%
256	2.85E-4	98.47%	5.02E-3	95.67%	7.30E-3	99.67%	7.23E-3	96.67%

^*^Baseline: calculate classification accuracy using the test RNAseq data. Additionally, compute the MMD score between the test RNAseq data and the train RNAseq data, which serves as the baseline criterion. ^*^GEMDiff(50%): only used 50% of train RNAseq data during the train phase. ^*^GEMDiff(30%): only used 30% of train RNAseq data during the train phase.

We further evaluated the GEMDiff model’s performance by training it with smaller subsets of the original training data, specifically 50% (394 samples) and 30% (264 samples). We observed that even with reduced training data, the synthesized data generated by the GEMDiff model still maintained a distribution similar to that of the test data (Supplemental Figs S3 and S4). We ran the argumentation experiment with WGAN [36] model (Supplemental Table A) as a comparison with GEMDiff. These results demonstrate the effectiveness of the GEMDiff model for data augmentation, as it generated real-like RNAseq data even with reduced training data samples.

### Identification of genes involved in transitions between normal and tumor gene expression states

To test if a given set of genes is involved in the gene expression state transition between labeled normal and tumor samples, we applied our workflow to normal (GTEx + TCGA normal) and breast tumor (TCGA) co-normalized bulk RNAseq samples. There are 78 724 known human genes (GENCODE v47). Unique combinations of genes lead to specific phenotype expression in an individual, and we sought to reduce the gene feature space without eliminating potentially causal genes. As a proof of principle, we targeted 50 expertly curated MSigDB Hallmark gene sets that “summarize and represent specific, well-defined biological states or processes and display coherent expression” (https://www.gsea-msigdb.org/gsea/msigdb/human/collections.jsp). These Hallmark genes contain genetic subsytems known to be involved in cellular processes altered in tumors and other biological systems, but these represent only a fraction of all possible coordinated gene expressions.

Table 2 shows more significant clustering potential between hallmark genes and random genes when the feature size is fewer than 64 (Student’s t-test *P* = 0.719). When the feature size is greater than 64, the silhouette score of random all experiments exceeds the random hallmark selection—we visualized the histogram in Supplemental Fig. S5. As in our previous work [11], and due to computational resource needs, we decided to use a gene feature size of 16 in perturbation experiments. Each Hallmark gene set was broken into randomly selected subsets of 16 genes without any overlap between gene sets, resulting in 423 Hallmark targeted gene subsets for analysis (Supplemental Table B). If there were not enough genes to reach 16 after random sorting, the remaining genes were ignored so that all targeted subsets were symmetrical at 16 genes. Next, we calculated the silhouette score based on UMAP representations of genes in an unbalanced sample experiment (i.e. all samples for both normal and tumor classes), comparing randomly selected gene subsets from the targeted gene sets. For the control group, 16 genes were randomly selected from all gene features 1000 times, yielding a mean silhouette score of 0.221 (stdev=0.120). The targeted group, which selected genes from the specific Hallmark gene set, shows a mean score of 0.245 (stdev=0.110). There was a significant difference between the random and targeted silhouette scores (Student’s t-test *P* = 0.0000031).

**Table 2:** Assess cluster potential with a range of gene feature sizes

**Experiments**	**Feature size**	**Sil.(mean)**	**Sil.(stdev)**
Random(ALL)	8	0.147	0.117
Random(HM)	8	0.181	0.117
Random(ALL)	**16**	**0.221**	**0.120**
Random(HM)	**16**	**0.245**	**0.110**
Random(ALL)	32	0.297	0.112
Random(HM)	32	0.325	0.093
Random(ALL)	64	0.389	0.087
Random(HM)	64	0.391	0.075
Random(ALL)	128	0.452	0.056
Random(HM)	128	0.439	0.055
Random(ALL)	256	0.485	0.037
Random(HM)	256	0.464	0.043

Random(ALL): random select genes from all $19\ 738$ genes; Random(HM): random select genes from Hallmark gene set.

**Figure 5:**
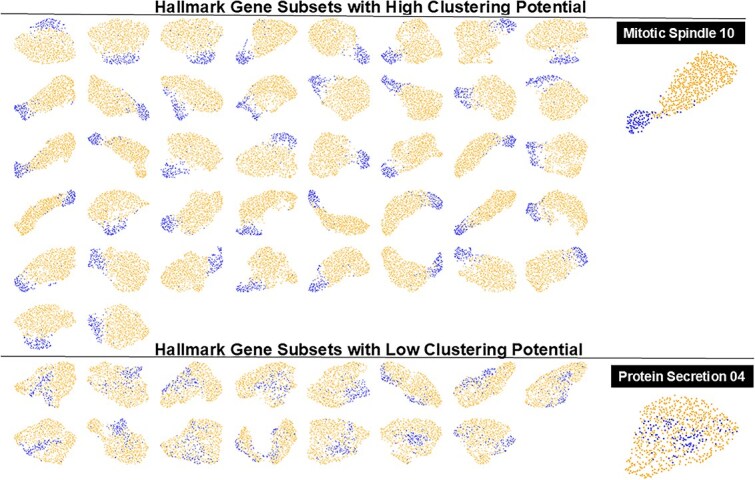
Hallmark gene subsets with high and low normal to tumor clustering potential: 243 Hallmark gene (n=16) subset GEMs were used to generate UMAP plots and silhouette scores for normal breast and breast tumor samples. Experiments with significant clustering or non-clustering are shown. The rightmost UMAP plots are the experiments with the highest and lowest silhouette scores.

We selected the well-clustered normal to tumor UMAP projections by identifying all gene sets with a silhouette score +/- one standard deviation from the silhouette mean (>0.401; <0.043). This circumscribed 43/423 gene subsets with high clustering potential and 16/423 gene subsets with low clustering potential (Fig. 5). The fraction of Hallmark set subsets that were significant are shown in Table 3.

**Table 3:** Hallmark gene subsets (n=16) with significant tumor versus normal sample clustering potential

**Geneset**	**Sil.(mean)**	**Sil.(stdev)**	**Sig. subsets**
PROTEIN_SECRETION	0.09	0.12	1/5
ALLOGRAFT_REJECTION	0.10	0.09	4/12
UNFOLDED_PROTEIN_RESPONSE	0.10	0.03	1/6
INTERFERON_GAMMA_RESPONSE	0.12	0.08	1/12
ESTROGEN_RESPONSE_EARLY	0.13	0.09	4/12
REACTIVE_OXYGEN_SPECIES_PATHWAY	0.14	0.11	0/2
INTERFERON_ALPHA_RESPONSE	0.15	0.06	0/5
OXIDATIVE_PHOSPHORYLATION	0.15	0.07	0/11
MYC_TARGETS_V1	0.15	0.07	0/12
ANDROGEN_RESPONSE	0.15	0.10	1/6
PEROXISOME	0.15	0.10	1/6
PANCREAS_BETA_CELLS	0.16	0.02	0/2
PI3K_AKT_MTOR_SIGNALING	0.18	0.11	0/6
ESTROGEN_RESPONSE_LATE	0.18	0.10	0/12
COMPLEMENT	0.20	0.10	1/12
MTORC1_SIGNALING	0.20	0.12	1/12
TGF_BETA_SIGNALING	0.20	0.06	0/3
APOPTOSIS	0.22	0.09	0/9
KRAS_SIGNALING_DN	0.22	0.09	0/12
UV_RESPONSE_UP	0.22	0.11	1/9
HEME_METABOLISM	0.22	0.08	0/11
IL6_JAK_STAT3_SIGNALING	0.23	0.08	0/5
P53_PATHWAY	0.23	0.07	0/11
ANGIOGENESIS	0.23	0.05	0/2
MYC_TARGETS_V2	0.23	0.11	0/3
INFLAMMATORY_RESPONSE	0.24	0.09	0/12
XENOBIOTIC_METABOLISM	0.25	0.08	1/12
DNA_REPAIR	0.26	0.06	0/9
EPITHELIAL_MESENCHYMAL_TRANSITION	0.26	0.10	1/12
BILE_ACID_METABOLISM	0.27	0.17	4/7
HYPOXIA	0.27	0.07	0/11
TNFA_SIGNALING_VIA_NFKB	0.28	0.08	1/12
APICAL_SURFACE	0.28	0.08	0/2
COAGULATION	0.29	0.06	0/8
GLYCOLYSIS	0.29	0.10	0/12
APICAL_JUNCTION	0.29	0.06	1/11
MYOGENESIS	0.29	0.09	1/12
FATTY_ACID_METABOLISM	0.29	0.11	1/9
ADIPOGENESIS	0.30	0.06	0/12
IL2_STAT5_SIGNALING	0.30	0.08	1/12
CHOLESTEROL_HOMEOSTASIS	0.30	0.09	1/4
KRAS_SIGNALING_UP	0.31	0.12	3/11
NOTCH_SIGNALING	0.33	0.12	1/2
WNT_BETA_CATENIN_SIGNALING	0.33	0.05	0/2
UV_RESPONSE_DN	0.33	0.08	2/8
HEDGEHOG_SIGNALING	0.34	0.00	0/2
SPERMATOGENESIS	0.35	0.11	4/8
G2M_CHECKPOINT	0.36	0.13	5/11
E2F_TARGETS	0.39	0.12	8/12
MITOTIC_SPINDLE	0.40	0.09	8/12

**Figure 6:**
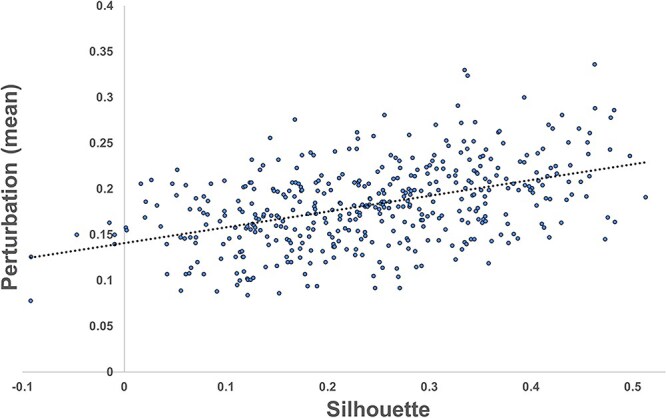
Correlation between GEMDiff transcriptional state perturbation and silhouette scores: the Hallmark gene subset perturbation mean of all genes in gene subsets versus silhouette scores.

To identify the genes associated with normal breast and breast tumor states, we ran the GEMDiff model on each of the Hallmark gene subsets where tumors were transitioned to normal samples. The average perturbation scores and directions for all genes can be found in Supplemental Table C. Interestingly, there was a positive correlation between gene perturbations and silhouette scores for the Hallmark gene subsets (Pearson’s r=0.450; Fig. 6). Next, we identified the 307 “core genes” that were the most perturbed in the transition from tumor back to normal states (+/- 2 SD from the mean). These genes can be found in Supplemental Table D.

In order to determine if the collective function of the 307 “core genes” was associated with breast cancer, we performed functional enrichment analysis. The top significantly enriched terms can be found in Table 4, and the full list of terms is located in Supplemental Table E (B&Y FDR <1e-10). Note the enrichment of breast cancer “Disease” terms.

**Table 4:** Top five “core gene” terms in each annotation category (B&Y FDR less than 1e-10)

**Category**	**ID**	**Name**	**Q**
Coexpression	M15664	ROSTY_CERVICAL_CANCER_PROLIFERATION_CLUSTER	6.19E-41
Coexpression	M41693	TRAVAGLINI_LUNG_PROLIFERATING_MACROPHAGE_CELL	2.92E-38
Coexpression	M3766	SOTIRIOU_BREAST_CANCER_GRADE_1_VS_3_UP	5.99E-38
Coexpression	M16010	KOBAYASHI_EGFR_SIGNALING_24HR_DN	2.32E-35
Coexpression	M18506	CROONQUIST_IL6_DEPRIVATION_DN	5.86E-34
Disease	C2239176	Liver carcinoma	2.10E-45
Disease	C4704874	Mammary carcinoma, human	1.27E-22
Disease	C1458155	Mammary neoplasms	1.27E-22
Disease	C1257931	Mammary neoplasms, human	1.27E-22
Disease	C0678222	Breast carcinoma	2.38E-22
GO: Biological Process	GO:0033993	Response to lipid	8.91E-16
GO: Biological Process	GO:0014070	Response to organic cyclic compound	1.16E-13
GO: Biological Process	GO:0043067	Regulation of programmed cell death	2.65E-13
GO: Biological Process	GO:0009725	Response to hormone	2.65E-13
GO: Biological Process	GO:1903047	Mitotic cell cycle process	4.21E-13
TFBS	M30019	HSD17B8_TARGET_GENES	1.30E-15

**Figure 7:**
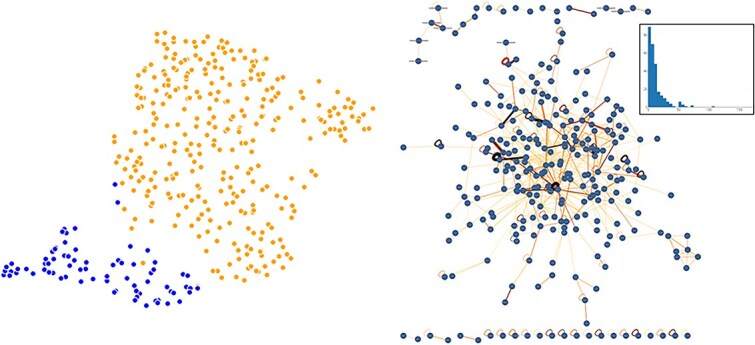
Breast cancer core genes: 307 core genes discovered with GEMDiff were used to (left) create a UMAP plot where blue dots indicate normal and orange dots represent breast tumor samples, and (right) an IntAct PPI network with the degree distribution shown in the box.

To identify physical interactions between the 307 “core genes,” we queried the IntAct database and built a PPI network, as shown in Fig. 7.

## Discussion

Here we introduce a workflow that filters gene features and applies GEMDiff to identify core genes during a simulated transition between gene expression states. We applied this workflow to normal breast and breast tumor sample groups and identified 307 breast cancer candidate genes. GEMDiff is notable for lacking task-specific hyper-parameter tuning, effectively training a single DM across tasks. Our method did not require restrictions on the loss function for data augmentation and perturbation. Due to GEMDiff’s robustness, we retained all samples even if there might be outliers. Commonly, outlier datasets were considered noise data and were dropped during the data pre-processing phase since they would reduce model performance. Our design concept is to keep all samples because we believe that these outliers also contain biological information. GEMDiff is robust enough to learn the biological relationships within the data while overcoming the side effect (decreasing model performance) brought by outlier data. This indicates that we did not need to sacrifice the integrity of the data to improve model performance. The generalized data pre-processing makes GEMDiff easy to generalize to other gene expression analysis tasks.

In addition, we demonstrate that small sample sizes will not affect the performance of GEMDiff. This was determined in an experiment using a relatively small number of samples ($67\%$) for training and validating the model performance on the remaining $33\%$ of samples, unlike the combination of training $80\%$ and testing $20\%$. We further trained the GEMDiff with a smaller data sample size – 50% of the original training data, (394 samples) and 30% of the original training data (264 samples), we observed that the synthesized data still maintained a similar distribution compared to the test data. Within the reasonable range of gene features and sample sizes, GEMDiff maintains good performance and does not encounter overfitting issues.

### GEMDiff stability

In contrast to models based on GANs, our GEMDiff model avoids the necessity of configuring a specific loss function to prevent model collapse. This robustness persists even when transitioning the model architecture from U-Net-based to transformer-based, eliminating the need to adjust parameters for weighting the loss function. In addition, we further discovered that one DM is sufficient to handle both data augmentation and gene perturbation tasks. This is a notable improvement over the Dual Diffusion Implicit Bridge approach, which requires the training of two separate DMs [31].

### GEMDiff scalability

GEMDiff is designed to scale effectively across a broad spectrum of gene features, with its capacity covering a substantial portion of the gene set. In our experiments, we tested gene feature sizes ranging from 8 to 256. Given that our dataset comprises only 1181 samples, we opted not to train the model on the full set of $19\ 738$ gene features for two reasons. First, we wanted to avoid overfitting and address the tendency for more than 64 random genes to generate sample clusters (Fig. 4). Second, we have observed this “background classification potential” in another study [11] where random sampling of more than 100 genes led to significant clustering, valid with silhouette score estimation (Supplemental Fig. S5).

### GEMDiff data augmentation

RNAseq data sometimes involve legal and ethical problems. DNA sequence data underlying RNAseq and some metadata (e.g. age, location) are clues to patient identity and often datasets are proprietary. Thus, it is often difficult to access data for research purposes without a legal and cybersecurity team. To circumvent these issues, rather than sharing the RNA-seq data directly, a well-trained GEMDiff could synthesize data closely resembling the original sequence data. For instance, the GEMDiff model encodes RNAseq data into a latent representation, as discussed in [31]. The well-trained model either retrieves the original-like RNA state from the latent representation or perturbs the RNA to another state, such as transforming tumor states back to normal to obtain real-like data. In principle, the simulated GEM data could be distributed as an open-source construct that could not be traced back to a patient but still retain the complex gene expression patterns underlying the trait.

### GEMDiff biommarker discovery

Tumors are derived from normal human tissue, and their gene pool is initiated from the host genome, which varies between individuals. Since there are currently 19 433 known protein-coding genes in humans, which is a subset of 78 724 total known gene models (Gencode v47; www.gencodegenes.org), both normal breast tissue and tumors are complex and highly dimensional. Once DNA mutations accumulate within this gene pool, tumor cells begin to adapt to and replicate within a dangerous host environment. Eventually, tumor cells, which may contain multiple clonal genetic backgrounds, begin to exhibit the eight hallmarks of cancer as they progress from a normal to a malignant state [37].

There are known driver genes that appear to play a role across cancer subtypes that originated from multiple tissue sources (e.g. TP53 is mutated in 29–35% of all tumors [38]). Many genes are mutated in fewer tumors, and there is no core mutated gene set for any given tumor subtype. It is the unique combination of aberrantly expressed genes in a tumor that drives the cancer phenotype. To underscore the complexity of “which genes cause cancer,” one study found that 15 233 out of 17 371 human genes have been associated with cancer [39]. Given this complexity and propensity for tumors to have unique sets of tumor drivers, new methods for circumscribing causal alleles are needed to understand and intervene in cancer biology.

In this study, we applied the GEMDiff computational workflow to 50 Hallmark gene sets that were grouped by prior knowledge of their coordinated function. Our assumption was that screening subsets of genes that are co-functional would improve the probability of hitting a relevant gene set. In addition, these gene sets are small enough that we could break them into sets of 16 genes to reduce the sample clustering that is seen with more than 64 random genes (Fig. 4). We have previously reported on this “background classification potential” effect [11]. We do not claim that 16 genes is the perfect subset of genes driving the expression state, but it is below the 64 gene threshold that random force cluster formation prevents the dissection of classification signal and noise.

We found that some Hallmark gene subsets were able to cluster the samples while others did not (Fig. 5). The Hallmark sets that did not cluster samples and are not associated with cancer (e.g. HEME_METABOLISM) behave similarly to gene sets that are associated with cancer (e.g. P53_PATHWAY, DNA_REPAIR). However, we found that these genes were effectively random gene sets that do not discriminate between sample groups. The highest clustering Hallmark gene sets with the top 4 average subset silhouette scores (high cluster potential) also have the largest number of significant subsets that are known to be involved in tumor biology (G2M_CHECKPOINT, E2F_TARGETS, MITOTIC_SPINDLE) and are not commonly associated with cancer (SPERMATOGENESIS, BILE_ACID_METABOLISM), indicating that these genes are non-randomly associated with the transition from tumor to normal states (Table 3).

After screening gene subsets (n=16) derived from co-functional Hallmark gene sets with high cluster potential, we identified 307 “core genes” that simulate the gene expression state between two sample groups: normal and tumor breast samples. Are these “core genes” truly involved in tumor transitions from the normal state, or are they simply the artifact of our simulation? There was a correlation (Pearson’s r=0.45) between perturbation and silhouette scores, indicating that the perturbed genes are correlated with clustering potential (Fig. 6). Further, the expression values match the perturbation results (Supplemental Tables F and G).

The functional enrichment results suggest that the core genes are involved in normal breast and breast tumor biology (Supplemental Table E): computational (cell cycle), disease (breast carcinoma), drug (17alpha-estradiol, 17-isoprogesterone), GO: biological process (epithelium development, mitotic cell cycle, mitotic nuclear division, positive regulation of cell population proliferation), TFBS (HSD17B8_TARGET_GENES). In addition, the “core gene” PPI network exhibits scale-free behavior (Fig. 7), suggesting that this is a realistic network of interacting genes at the protein level. While not definitive, the screening of the 50 Hallmark genes did reveal a candidate genetic subsystem that could be driving tumorigenesis.

How applicable is GEMDiff to other tumor sub-types? To test this, we applied GEMDiff to normal and tumor thyroid GEMs obtained from the same source as the breast dataset. GEMDiff identified 326 significantly perturbed thyroid “core genes” (Supplemental Tables H and I). Similar to the breast “core gene” PPI network, the thyroid PPI network was scale-free with similar topology (Supplemental Fig. S6). Functional enrichment analysis of the 326 thyroid “core genes” detected thyroid cancer enrichment co-expression (DELYS_THYROID_CANCER_UP, DELYS_THYROID_CANCER_DN) and ToppCell Atlas (TCGA-Thryoid-Primary_Tumor-Thyroid_Papillary_Carcinoma-Classical) groups (Supplemental Table J). These results suggest that GEMDiff will function on other cancer subtypes and should, in principle, be applicable to other complex phenotypes.

## Conclusion

Here we present a well-defined and simple diffusion workflow (GEMDiff) that augments RNAseq datasets and bridges gene expression perturbations between gene expression states (i.e. normal to tumor transitions). GEMDiff has two key contributions. First, we integrate the workflow with UMAP and silhouette scores to automatically filter the gene set with high cluster potential. Furthermore, GEMDiff integrates gene augmentation and gene perturbation in a single DM, as it no longer requires a specific loss function for different tasks, identifying biomarkers for breast and thyroid cancer transitions. We ran a series of experiments that proved the practical value of GEMDiff in cancer biomarker discovery. Note that this was a case study using TCGA and GTEx breast bulk RNAseq datasets, but the approach can be applied to any gene expression transitions measured at the bulk or single-cell RNAseq levels, even beyond humans.

**Key Points**

- GEMDiff offers a simplified workflow that includes data pre-processing, cluster potential estimation, gene expression augmentation and perturbation, and biological validation.
- GEMDiff incorporates a transformer-based model to better capture broad gene feature relationships, making it more effective at revealing intricate gene interrelationships than traditional convolutional networks.
- We have focused on enhancing the stability and scalability of GEMDiff, transforming it into a generalized workflow suitable for various gene expression tasks, as it no longer requires a specific loss function for different tasks.
- GEMDiff operates an ODE process to revert the tumor gene expression back to normal as a form of entropy-regularized optimal transport. It visualizes these procedures with UMAP plots, introducing a novel perspective to gene expression analysis.
- GEMDiff discovered 307 core breast cancer Hallmark genes and 326 core thyroid cancer Hallmark genes.

## Supplementary Material

Ai_etal_SupplementalFigures_v12_bbaf093

Ai_etal_SupplementalTables_v10_bbaf093

## Data Availability

GEMDiff source code: https://github.com/xai990/GEMDiff.git OMIM database: https://www.omim.org/statistics/geneMap MSigDB dataset: https://www.gsea-msigdb.org/gsea/msigdb/human/collections.jsp

## References

[1] Sung H, Ferlay J, Siegel RL. et al. Global cancer statistics 2020: GLOBOCAN estimates of incidence and mortality worldwide for 36 cancers in 185 countries. *CA Cancer J Clin* 2021;71:209–49. 33538338 10.3322/caac.21660

[2] Nolan E, Lindeman GJ, Visvader JE. Deciphering breast cancer: From biology to the clinic. *Cell* 2023;186:1708–28. 10.1016/j.cell.2023.01.040 36931265

[3] Turashvili G, Brogi E. Tumor heterogeneity in breast cancer. *Front Med* 2017;4:227. 10.3389/fmed.2017.00227 PMC5727049 29276709

[4] Gavriilidis GI, Vasileiou V, Orfanou A. et al. A mini-review on perturbation modelling across single-cell omic modalities. *Comput Struct Biotechnol J* 2024;23:1886–96. 10.1016/j.csbj.2024.04.058 38721585 PMC11076269

[5] Colin Targonski M, Bender R, Shealy BT. et al. Cellular state transformations using deep learning for precision medicine applications. *Patterns* 2020;1:100087. 10.1016/j.patter.2020.100087 33205131 PMC7660411

[6] Hang Y, Burns J, Shealy BT. et al. Identification of condition-specific regulatory mechanisms in normal and cancerous human lung tissue. *BMC Genomics* 2022;23:350. 10.1186/s12864-022-08591-9 PMC9077899 35524179

[7] Nelligan NM, Reed Bender M, Alex F. et al. Simulating the restoration of normal gene expression from different thyroid cancer stages using deep learning. *BMC Cancer* 2022;22:612. 10.1186/s12885-022-09704-z PMC9166476 35659616

[8] Ai X, Smith MC, Feltus FA. Generative adversarial networks applied to gene expression analysis: an interdisciplinary perspective. *Comput Syst Oncol* 2023;3:e1050.

[9] Viñas R, Andrés-Terré H, Liò P. et al. Adversarial generation of gene expression data. *Bioinformatics* 2022;38:730–7. 10.1093/bioinformatics/btab035 33471074 PMC8756177

[10] Wang L, Yan X, You Z-H. et al. SGANRDA: semi-supervised generative adversarial networks for predicting circRNA–disease associations. *Brief Bioinform* 2021;22:bbab028. 33734296 10.1093/bib/bbab028

[11] Targonski CA, Shearer CA, Shealy BT. et al. Uncovering biomarker genes with enriched classification potential from hallmark gene sets. *Sci Rep* 2019;9:1–10. 10.1038/s41598-019-46059-1 31278367 PMC6611793

[12] Lee K, Kim T, Cheon M. et al. Unveiling oasis family as a key player in hypoxia–ischemia cases induced by cocaine using generative adversarial networks. *Sci Rep* 2022;12:1–15. 10.1038/s41598-022-10772-1 35469040 PMC9038918

[13] Wei X, Dong J, Wang F. scPreGAN, a deep generative model for predicting the response of single-cell expression to perturbation. *Bioinformatics* 2022;38:3377–84. 10.1093/bioinformatics/btac357 35639705

[14] Ho J, Jain A, Abbeel P. Denoising diffusion probabilistic models. *Adv Neural Inf Process Syst* 2020;33:6840–51.

[15] Song J, Meng C, Ermon S. Denoising diffusion implicit models arXiv preprint arXiv:2010.02502. 2020.

[16] Song Y, J Sohl-Dickstein, DP Kingma, et al. Score-based generative modeling through stochastic differential equations arXiv preprint arXiv:2011.13456, 2020.

[17] Peng W, Huabin D, Yan Y. et al. Guided diffusion for molecular generation with interaction prompt. *Brief Bioinform* 2024;25:bbae174. 10.1093/bib/bbae174 PMC11033848 38647154

[18] Li K, Li J, Tao Y. et al. stDiff: a diffusion model for imputing spatial transcriptomics through single-cell transcriptomics. *Brief Bioinform* 2024;25:bbae171. 38628114 10.1093/bib/bbae171 PMC11021815

[19] Gruver N, Stanton S, Frey N. et al. Protein design with guided discrete diffusion. *Adv Neural Inf Process Syst* 2024;36:12489–517.

[20] Zhang Z, Liu L. scIDPMs: single-cell RNA-seq imputation using diffusion probabilistic models. IEEE Journal of Biomedical and Health Informatics 2024. 10.1109/JBHI.2024.3430554. 39093668

[21] Wang Y, Chen Q, Shao H. et al. Generating bulk RNA-seq gene expression data based on generative deep learning models and utilizing it for data augmentation. *Comput Biol Med* 2024;169:107828. 10.1016/j.compbiomed.2023.107828 38101117

[22] Lacan A, Andre R, Sebag M. et al. In silico generation of gene expression profiles using diffusion models. *bioRxiv* 2024;2024–04. 10.1186/s12859-026-06470-8 PMC13418420 42226153

[23] Weitzel JN, Lagos V, Blazer KR. et al. Prevalence of BRCA mutations and founder effect in high-risk hispanic families. *Cancer Epidemiol Biomarkers Prev* 2005;14:1666–71. 10.1158/1055-9965.EPI-05-0072 16030099

[24] Ronneberger O, Fischer P, Brox T. U-net: convolutional networks for biomedical image segmentation. In Medical Image Computing and Computer-assisted Intervention–MICCAI 2015: 18th International Conference, Munich, Germany, October 5–9, 2015, proceedings, part III 18, pp. 234–41. Springer, 2015.

[25] Vaswani A, Shazeer N, Parmar N. et al. Attention is all you need. In: Advances in Neural Information Processing Systems, pp. 5998–6008, 2017.

[26] McInnes L, Healy J, Melville J. UMAP: uniform manifold approximation and projection for dimension reduction arXiv preprint arXiv:1802.03426. 2018.

[27] Wang Q, Armenia J, Zhang C. et al. Unifying cancer and normal RNA sequencing data from different sources. *Scientific Data* 2018;5:1–8. 29664468 10.1038/sdata.2018.61 PMC5903355

[28] Weinstein JN, Collisson EA, Mills GB. et al. The cancer genome atlas pan-cancer analysis project. *Nat Genet* 2013;45:1113–20. 10.1038/ng.2764 24071849 PMC3919969

[29] Lonsdale J, Thomas J, Salvatore M. et al. The genotype-tissue expression (GTEx) project. *Nat Genet* 2013;45:580–5. 10.1038/ng.2653 23715323 PMC4010069

[30] Rousseeuw PJ . Silhouettes: a graphical aid to the interpretation and validation of cluster analysis. *J Comput Appl Math* 1987;20:53–65. 10.1016/0377-0427(87)90125-7

[31] Su X, Song J, Meng C. et al. Dual diffusion implicit bridges for image-to-image translation arXiv preprint arXiv:2203.08382. 2022.

[32] Gretton A, Borgwardt KM, Rasch MJ. et al. A kernel two-sample test. *J Mach Learn Res* 2012;13:723–73.

[33] Suthaharan S . Support vector machine. Machine Learning Models and Algorithms for Big Data Classification. Integrated Series in Information Systems, vol 36, pp. 207–35. Boston, MA: Springer, 2016. 10.1007/978-1-4899-7641-3_9.

[34] Chen J, Bardes EE, Aronow BJ. et al. ToppGene suite for gene list enrichment analysis and candidate gene prioritization. *Nucleic Acids Res* 2009;37:W305–11. 10.1093/nar/gkp427 19465376 PMC2703978

[35] Theis L, van den Oord A, Bethge M. A note on the evaluation of generative models arXiv preprint arXiv:1511.01844. 2015.

[36] Arjovsky M, Chintala S, Bottou L. Wasserstein generative adversarial networks. In:*International Conference on Machine Learning*, pp. 214–23. Sydney, Australia: PMLR, 2017.

[37] Hanahan D . Hallmarks of cancer: new dimensions. *Cancer Discov* 2022;12:31–46. 10.1158/2159-8290.CD-21-1059 35022204

[38] Bouaoun L, Sonkin D, Ardin M. et al. TP53 variations in human cancers: new lessons from the IARC TP53 database and genomics data. *Hum Mutat* 2016;37:865–76. 10.1002/humu.23035 27328919

[39] De Magalhães JP . Every gene can (and possibly will) be associated with cancer. *Trends Genet* 2022;38:216–7. 10.1016/j.tig.2021.09.005 34756472

