# Supplementary material for: GEMDiff: a diffusion workflow bridges between normal and tumor gene expression states: a breast cancer case study
Source: Brief Bioinform. 2025 Mar 11;26(2):bbaf093. doi: 10.1093/bib/bbaf093 (PMC11894803; doi:10.1093/bib/bbaf093)
Supplement: Ai_etal_SupplementalFigures_v12_bbaf093 [file ai_etal_supplementalfigures_v12_bbaf093.pptx]

## Slide 1
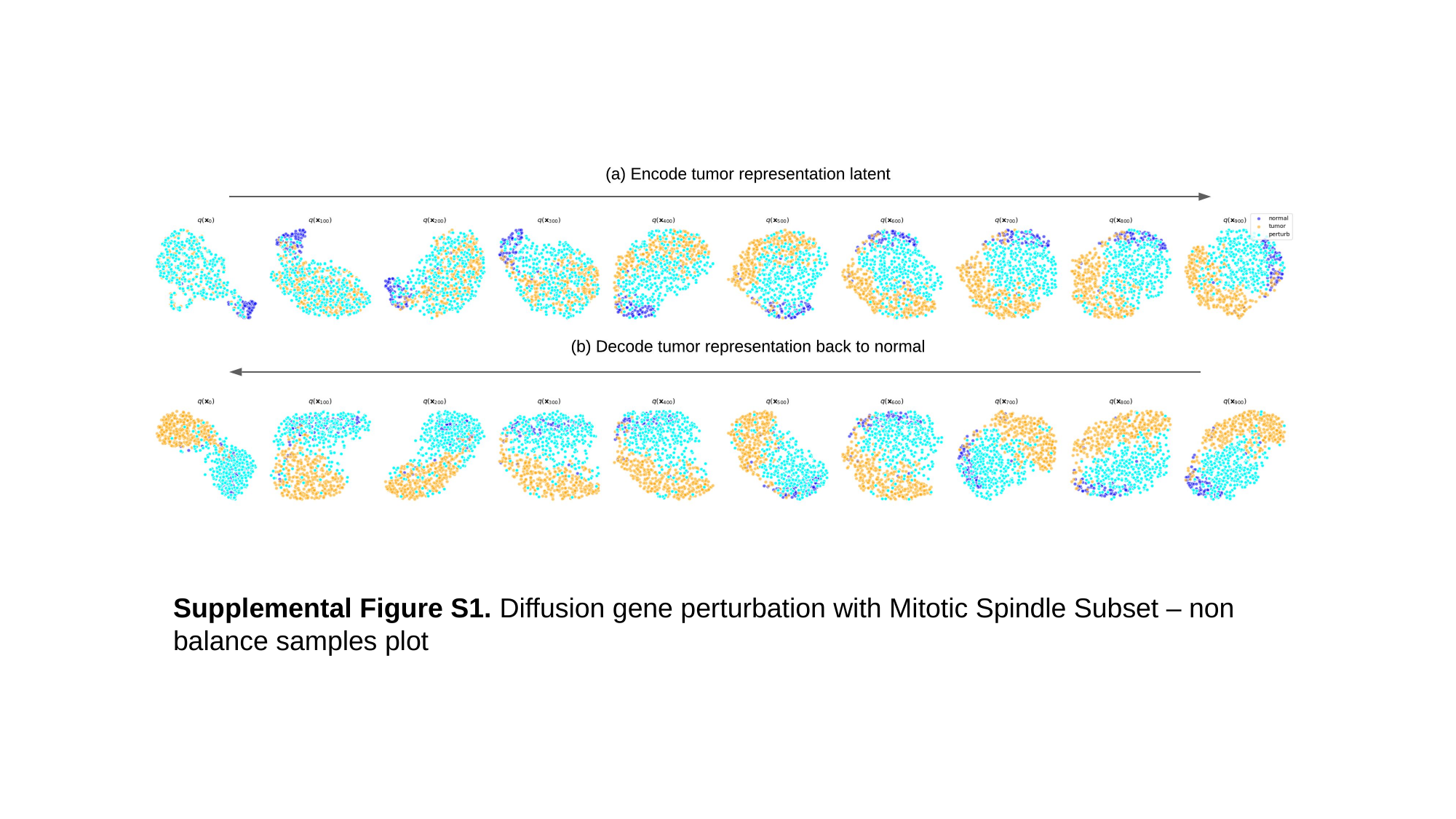

Supplemental Figure S1. Diffusion gene perturbation with Mitotic Spindle Subset – non balance samples plot

## Slide 2
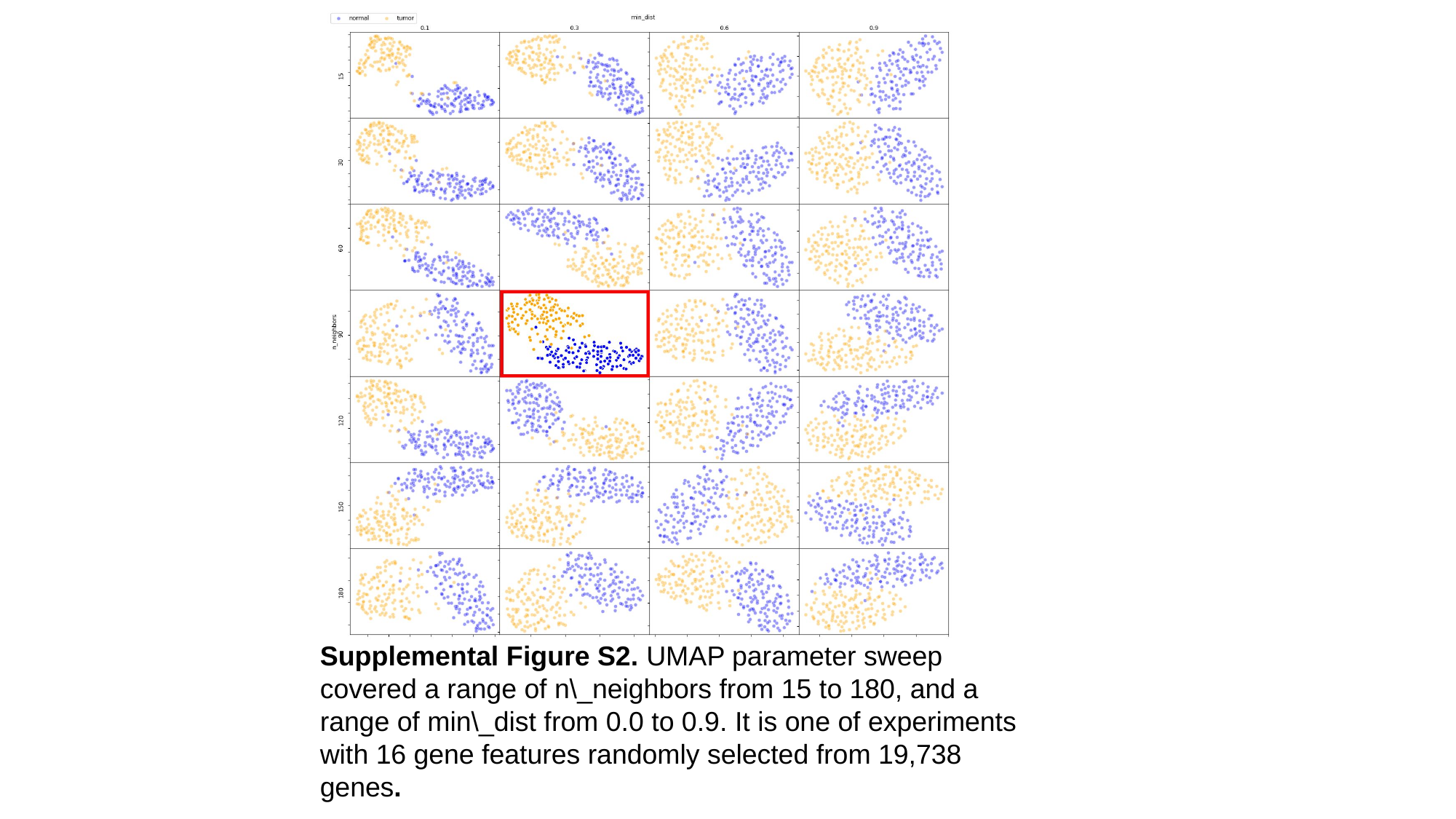

Supplemental Figure S2. UMAP parameter sweep covered a range of n\_neighbors from 15 to 180, and a range of min\_dist from 0.0 to 0.9. It is one of experiments with 16 gene features randomly selected from 19,738 genes.

## Slide 3
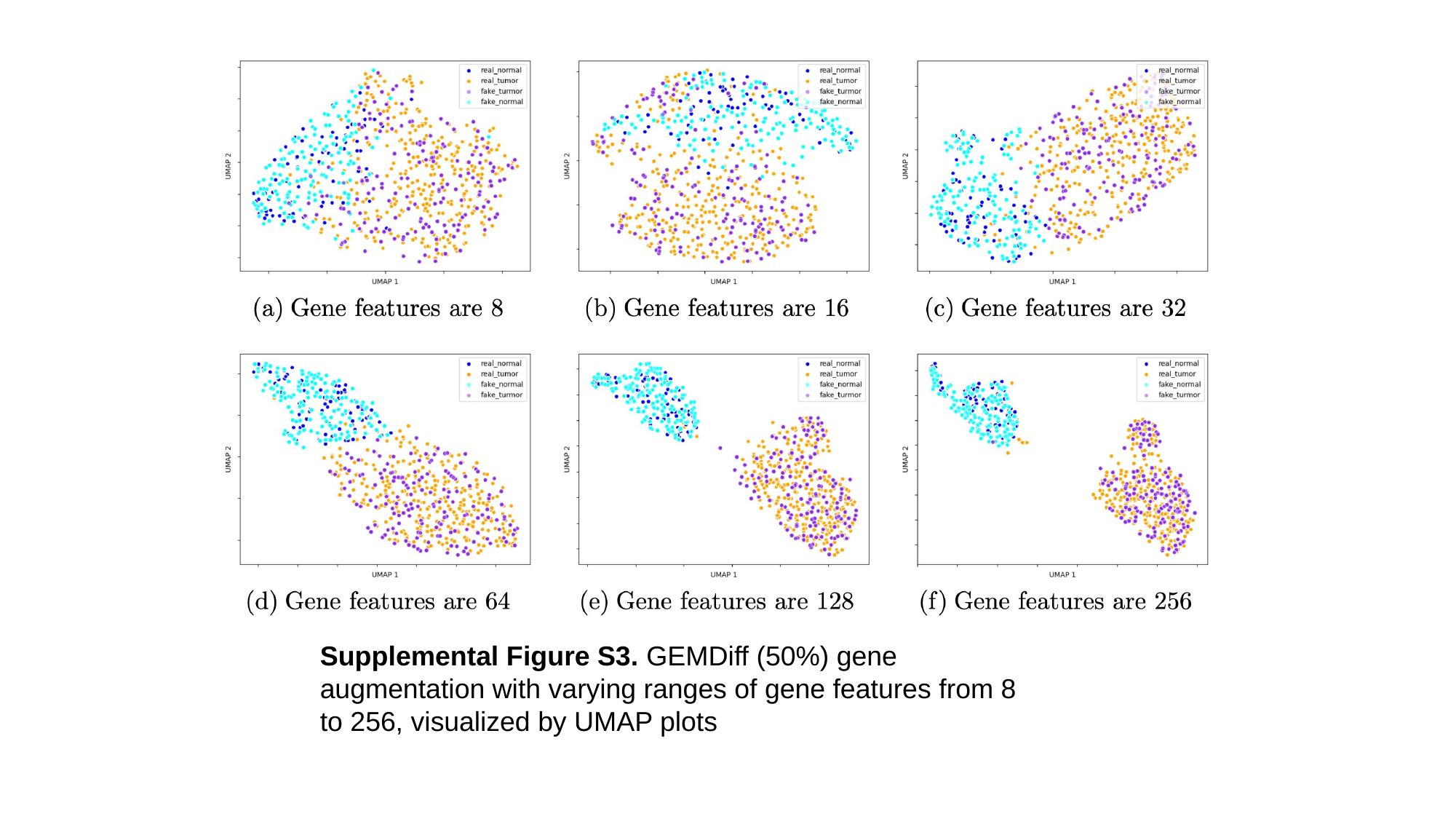

Supplemental Figure S3. GEMDiff (50%) gene augmentation with varying ranges of gene features from 8 to 256, visualized by UMAP plots

## Slide 4
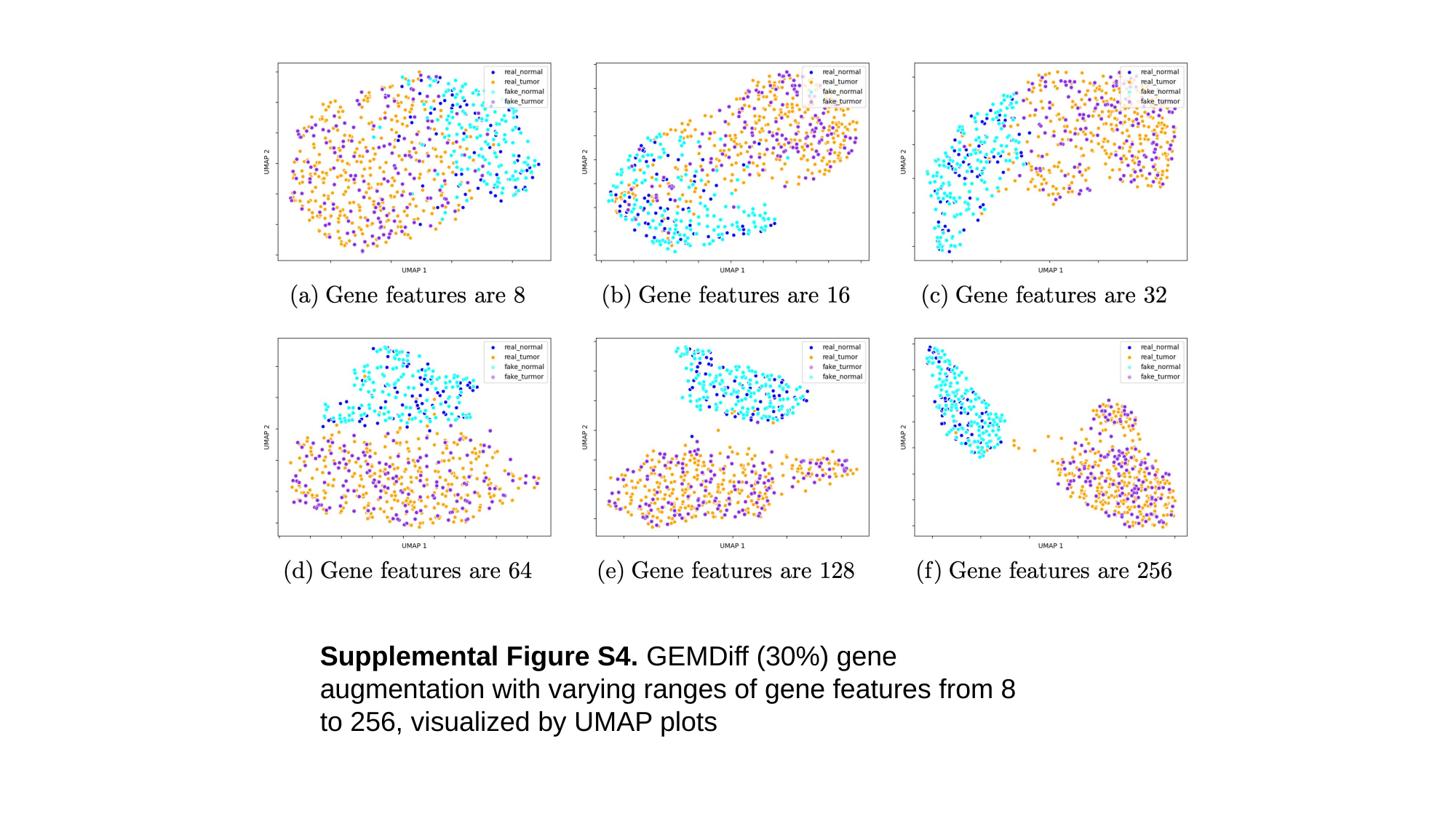

Supplemental Figure S4. GEMDiff (30%) gene augmentation with varying ranges of gene features from 8 to 256, visualized by UMAP plots

## Slide 5
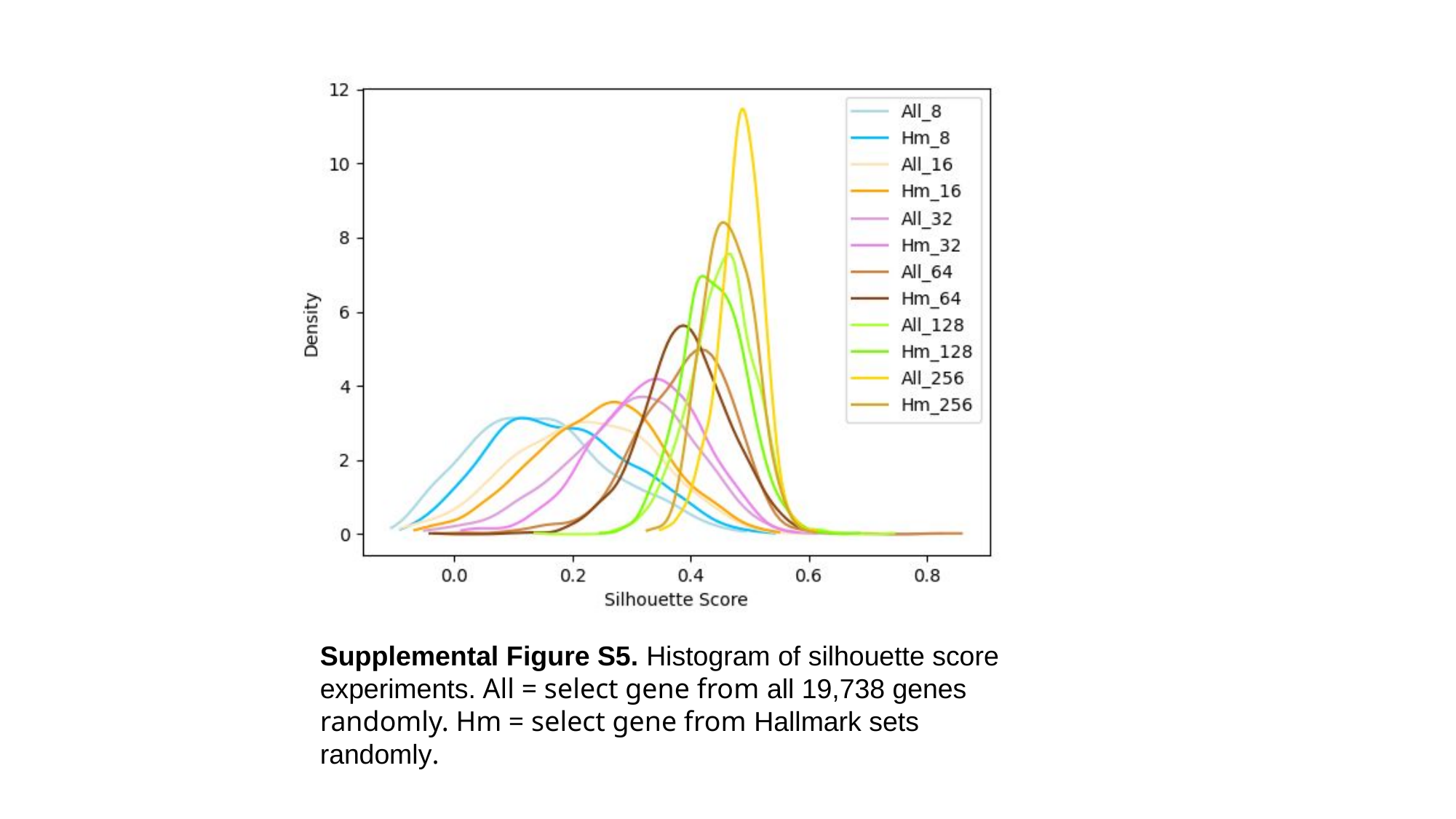

Supplemental Figure S5. Histogram of silhouette score experiments. All = select gene from all 19,738 genes randomly. Hm = select gene from Hallmark sets randomly.

## Slide 6
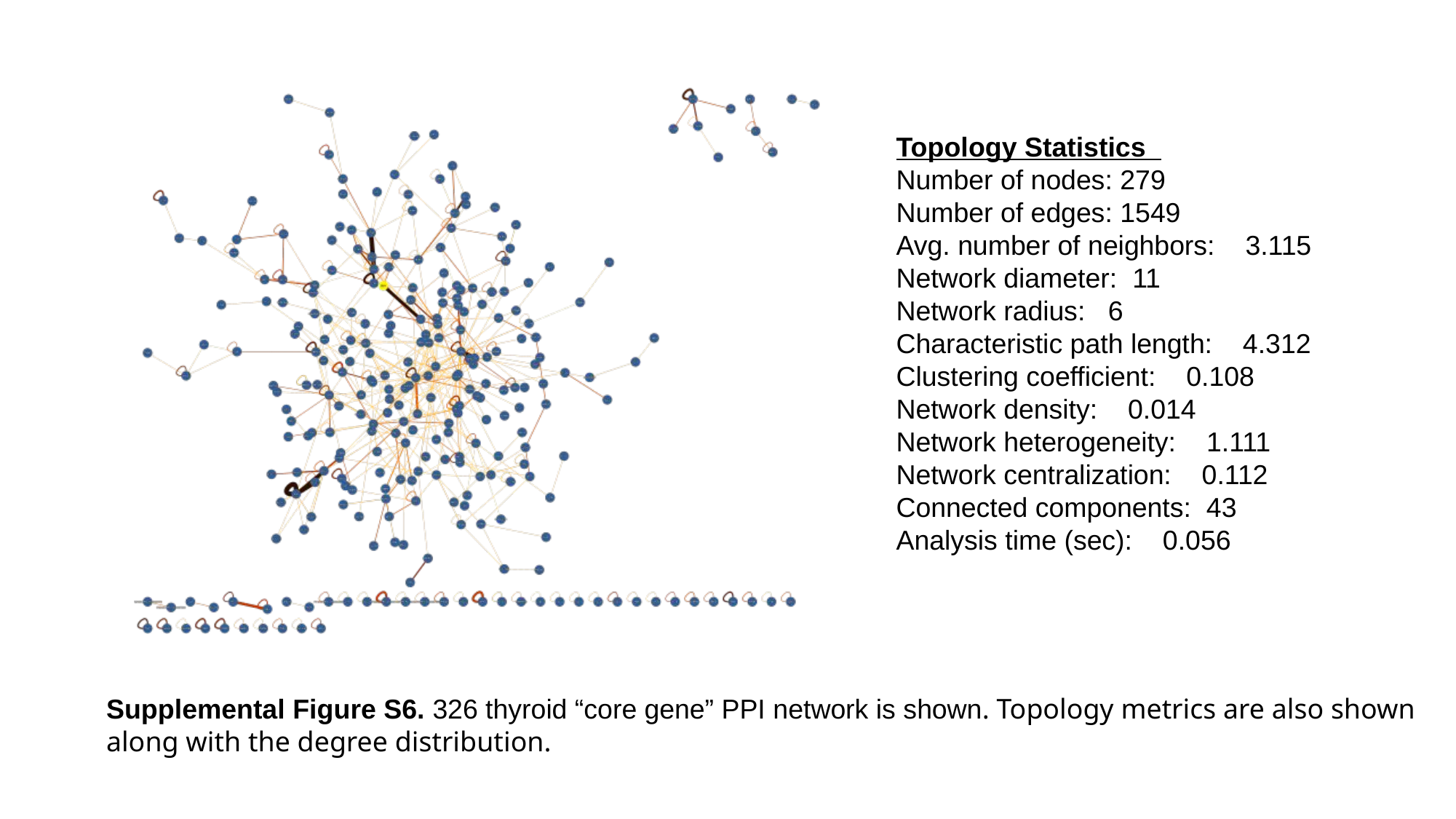

Topology Statistics
Number of nodes: 279
Number of edges: 1549
Avg. number of neighbors: 3.115
Network diameter: 11
Network radius: 6
Characteristic path length: 4.312
Clustering coefficient: 0.108
Network density: 0.014
Network heterogeneity: 1.111
Network centralization: 0.112
Connected components: 43
Analysis time (sec): 0.056
Supplemental Figure S6. 326 thyroid “core gene” PPI network is shown. Topology metrics are also shown along with the degree distribution.
